# Treatment outcomes of alternating chemoradiotherapy for nasopharyngeal carcinoma: a single-center safety and efficacy study

**DOI:** 10.1016/j.bjorl.2022.12.004

**Published:** 2022-12-28

**Authors:** Kenzo Ohara, Miki Takahara, Takumi Kumai, Masaaki Yamashina, Kan Kishibe, Akihiro Katada, Tatsuya Hayashi

**Affiliations:** a Asahikawa Medical University, Department of Otolaryngology-Head and Neck Surgery, Asahikawa, Japan; b Asahikawa Medical University, Department of Innovative Head & Neck Cancer Research and Treatment (IHNCRT), Asahikawa, Japan; c Asahikawa Medical University, Department of Radiology, Asahikawa, Japan

**Keywords:** Alternating chemoradiotherapy, Nasopharyngeal carcinoma, Cisplatin, 5-fluorouracil

## Abstract

**Objective:**

To evaluate the efficacy and safety of Alternating Chemoradiotherapy (ACRT) using cisplatin and 5-Fluorouracil (5-FU) in patients with nasopharyngeal carcinoma.

**Methods:**

This was a retrospective study in which patients’ clinical records were reviewed to identify patients with a new diagnosis of nasopharyngeal carcinoma at our institution between January 2005 and January 2019. Thirty-seven eligible patients were identified; of these, the clinical details of 27 patients treated with ACRT were evaluated. Patient outcomes, including overall survival and progression-free survival, and adverse events were assessed.

**Results:**

Of these initial 37 patients, 1, 10, 13, 10, and 3 were staged as I, II, III, IVA, and IVB, respectively, as defined by the 8th edition of the TNM classification system. Twenty-seven patients received ACRT comprising sequential administration of chemotherapy, radiotherapy (wide field), chemotherapy, radiotherapy (shrinking field), and chemotherapy. The 5-year overall survival and progression-free survival rates were 83.7% and 88.9%, respectively. Treatment compliance was 93%, which is comparable to that of previous reports.

**Conclusion:**

ACRT using cisplating and 5-fluorouracil was well tolerated with acceptable efficacy.

**Level of Evidence:**

IVa

## Highlights

• The safety and efficacy of alternating chemoradiotherapy using cisplatin and 5-fluorouracil were retrospectively examined in nasopharyngeal carcinoma patients.
• The overall comlete response rate was 93% with preserving renal function.
• The 5-year overall survival and progression-free survival rates were 83.7% and 88.9%, respectively.
• Treatment compliance, the 5-year overall survival rate and the 5-year overall survival rate are comparable to that of previous reports.
• Alternating chemoradiotherapy using cisplatin and 5-fluorouracil in nasopharyngeal carcinoma patients was well tolerated with acceptable efficacy.

## Introduction

Nasopharyngeal Carcinoma (NPC) is an epithelial malignancy with a rare incidence in the Western world; however a higher incidence of NPC is reported in East and Southeast China. In general, surgical resection is not the first treatment choice because of the anatomical characteristics of the nasopharynx; therefore, the primary treatment modality for NPC is chemoradiotherapy. Although platinum-based Concurrent Chemoradiotherapy (CCRT) is often used as a standard therapy, the completion ratio is poor due to severe adverse events.^1^

Several reports have described the efficacy of induction chemotherapy, but the results remain controversial.^2^, ^3^, ^4^, ^5^, ^6^ Alternating Chemoradiotherapy (ACRT) was originally developed by Fuwa et al.^7^ to reduce adverse events and improve the completion rate without reducing clinical outcomes. In this study, we assessed the efficacy and safety of ACRT for NPC at our institution.

## Methods

### Patients

Between January 2005 and January 2019, 37 Asian patients were newly diagnosed with NPC at our institution. Of these, 27 patients received ACRT as part of their standard care. The remaining patients with early-stage NPC, distant metastasis, or impaired renal condition received radiotherapy only, CCRT, or palliative treatments. Patients were staged according to the TNM classification by the Union for International Cancer Control (UICC) staging system. The exclusion criteria were patients with histological proof of T1, N0, M0 carcinoma of the nasopharynx. The following pretreatment clinical data were evaluated in all patients: medical history; physical examination findings; laryngoscopy and esophagoscopy results; and diagnostic imaging results (Computed Tomography [CT], Magnetic Resonance Imaging [MRI], and/or F-fluorodeoxy Glucose Positron Emission Tomography [FDG-PET]). Since FDG-PET became available after November 2005 at our institution, four patients who began treatment prior to November 2005 did not undergo FDG-PET scans for initial staging.

All 37 patients were evaluated by head and neck surgeons and radiation oncologists before treatment initiation. The study protocol was approved by the Institutional Review Board of our institution (approval number 20054), and all patients provided written informed consent before initiation of treatment. This study was conducted in accordance with the tenets of the Declaration of Helsinki.

### Alternating chemoradiotherapy protocol

**Figure 1:**
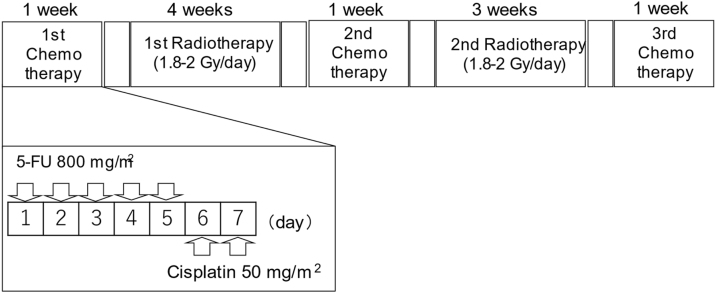
Study design of alternating chemoradiotherapy. During the course of chemotherapy (one-week period; three cycles), patients were irradiated at 2- or 3-day intervals of chemotherapy. 5-fluorouracil (5-FU) (800 mg/m^2^/day) was intravenously administered continuously for 24 h from days 1 to 5. Cisplatin (50 mg/m^2^/day) was intravenously administered continuously for 24 h from days 6 to 7. The same chemotherapy regimen was used in all three cycles of chemotherapy. Radiotherapy was delivered to the nasopharynx and whole neck, at 1.8–2 Gy/fraction, 5 fractions/week, until a dose of 50 Gy. Subsequent radiotherapy was delivered to the primary region and the remaining lymph nodes. The total dose during the study period was 68.4–73.4 Gy in 35–40 fractions.

ACRT was administered based on a modification of the original method by Fuwa et al.,^7^ which was published in 2007 (Fig. 1). Briefly, a course of chemotherapy was performed in three cycles (one week per cycle). Between each chemotherapy cycle, patients were administered two courses of radiotherapy. The interval between chemotherapy and radiotherapy was two or three days.

Regarding the chemotherapy protocol, patients were continuosuly intravenously administered 5-FU at a dose of 800 mg/m^2^/day for 5 days (day 1–5) and then Cisplatin (CDDP) at a dose of 50 mg/m^2^/day for 2 days (day 6–7). The total doses of 5-FU and CDDP during three cycle of chemotherapy were 12,000 mg/m^2^ and 300 mg/m^2^, respectively.

Regarding radiotherapy, the first 19 patients were treated with fixed multiport irradiation at an irradiation dosage of 68.4–73.4 Gy in 36–40 fractions until June 2014. Between June 2014 and January 2015, three patients were treated with a combination of fixed multiport irradiation and Intensity-Modulated Radiation Therapy (IMRT) at an irradiation dosage of 70.4–72.4 Gy in 38–39 fractions. After March 2015, five patients were treated with IMRT at an irradiation dosage of 70 Gy in 35 fractions. The initial irradiation field overlapping first and second radiotherapy courses was the whole neck from the skull base to the supraclavicular fossa at an irradiation dosage of 50 Gy, with a rest period of 2 weeks, during which patients underwent the second chemotherapy cycle. Subsequently, a shrinking irradiation field was used, which was administered to the primary lesion and the lymph nodes. The total dose for the entire radiotherapy was 68.4–73.4 Gy in 35–40 fractions.

### Evaluation of treatment response and toxicity

Pharyngeal endoscopic examination during and after treatment was used to evaluate the anti-tumor effects of ACRT. We primarily focused on the results of FDG-PET scans performed three months after the completion of ACRT. The assessments of response were also performed using MRI, CT, or palpation. Tumor response rate was evaluated using the Response Evaluation Criteria in Solid Tumors (RECIST).^8^ All adverse events encountered during therapy were evaluated according to the Common Terminology Criteria for Adverse Events (CTCAE; version 5.0).

### Measurement of plasma Epstein-Barr virus (EBV)-DNA viral load and detection of EBV-DNA in tissues by EB-encoded small RNA 1 (EBER)

We analyzed Epstein-Barr Virus (EBV)-DNA in plasma specimens of the 27 patients before and three months after ACRT. Histological samples were examined to determine whether cancer cells were positive for EB-encoded small RNA 1 (EBER).

### Ethical consideration

All procedures performed in this studies involving human participants follows the 1964 Helsinki declaration and its later amendments or comparable ethical standards. We obtained written informed consent from all individual participants included in the study.

### Statistical analyses

All statistical analyses were performed using GraphPad Prism version 9 (GraphPad Software, San Diego, CA, USA). Overall Survival (OS) and Progression-Free Survival (PFS) were calculated using the Kaplan-Meier method and compared using the log-rank test.

## Results

### Patient characteristics

The characteristics of the 27 patients who received ACRT and 10 patients who did not receive ACRT are shown in Table 1 and Supplemental Table 1, respectively. Of the 27 patients who underwent ACRT, 21 were men and six were women. The patients’ ages ranged from 22 to 74 years (median, 57 years). Histological examination revealed that 23 and 4 cases of NPC were non-keratinizing and keratinizing subtypes, respectively. In addition, EBER in situ hybridization was positive in 17/25 cases (68%). Two patients were not assessed for EBER status because EBER status evaluation did not become available at our institution until after 2007. Of the 27 patients who received ACRT, 5, 13, 8, and 1 patient(s) were staged as II, III, IVA, and IVB, respectively (Table 1, Table 2).

**Table 1:** Clinical characteristics of the patients (n = 27).

Characteristics	Nº of patients
Age, year	
Range	22‒72
Median	57
Mean	53.8
Stage	
II	5
III	13
IVA	8
IVB	1
Sex	
Male	21
Fernale	6
Histology	
Non-keratinizing	23
Keratinizing	4
EBER ISH	
Positive	17
Negative	8
NP	2

NP, not performed.

**Table 2:** T and N stage (n = 27).

T classification	Nº of patient’s by N classification				Total
	0	1	2	3	
1	0	2	4	0	6
2	2	1	2	1	6
3	0	5	2	1	8
4	2	2	2	1	7
Total	4	10	10	3	27

### Compliance

Of the 27 patients, 25 (93%) completed the ACRT regimen as planned without interruption. One patient could not complete the third chemotherapy cycle, and the other patient received a 50% reduced dose of the third chemotherapy cycle, both due to renal failure.

### Adverse events

The occurrence and incidence of hematological and non-hematological toxic events associated with ACRT are shown in Table 3. No treatment-related deaths occurred. Grade 3–4 hematologic adverse events were observed in 62% of patients. Grade 3–4 mucositis was observed in 70% of patients. No grade 3–4 renal toxicities were observed.

**Table 3:** Toxicity (n = 27).

Toxicity	Nº of patients by toxicity grade			
	1	2	3	4
Hemoglobin	5	12	5	0
Leukocytes	2	7	14	2
Platelets	11	2	1	2
Mucositis	3	4	18	1
Nauseas/vomiting	8	7	10	0
Renal impairment	2	2	0	0
Hepatic impairment	9	2	0	0
Diarrhea	3	1	1	0

### Treatment response and pattern of recurrences

**Figure 2:**
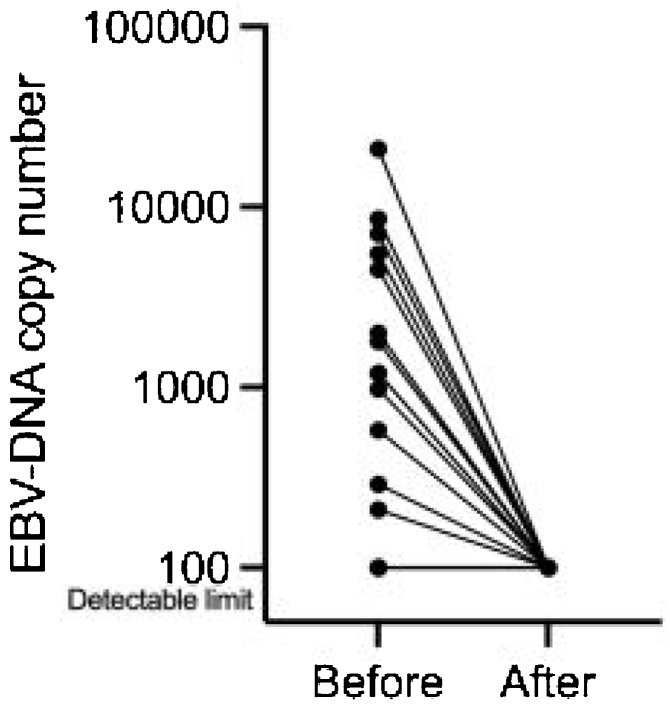
Plasma EBV-DNA level before and after ACRT. (a) Plasma EBV-DNA before ACRT, (b) Plasma EBV-DNA three months after ACRT. Measurable EBV-DNA were detected in 13 of 27 cases. All 13 cases showed a decrease in EBV-DNA three months after ACRT.

Three months after ACRT completion, all patients achieved complete responses at the primary site. The plasma EBV-DNA viral load in detectable 13 patients decreased to undetectable levels (Fig. 2).

Although neck dissection surgeries were performed after ACRT in two patients, pathological examination revealed no viable tumor cells in either case.

As of February 2021, 21 of the 27 patients were alive, and all 21 living patients were tumor-free. One patient developed recurrence at the primary site, and two patients developed distant metastases (lung or liver). Three patients died of other causes, comprising pneumonia, arrhythmia, and amyotrophic lateral sclerosis.

### Survival outcomes and factors related to survival

**Figure 3:**
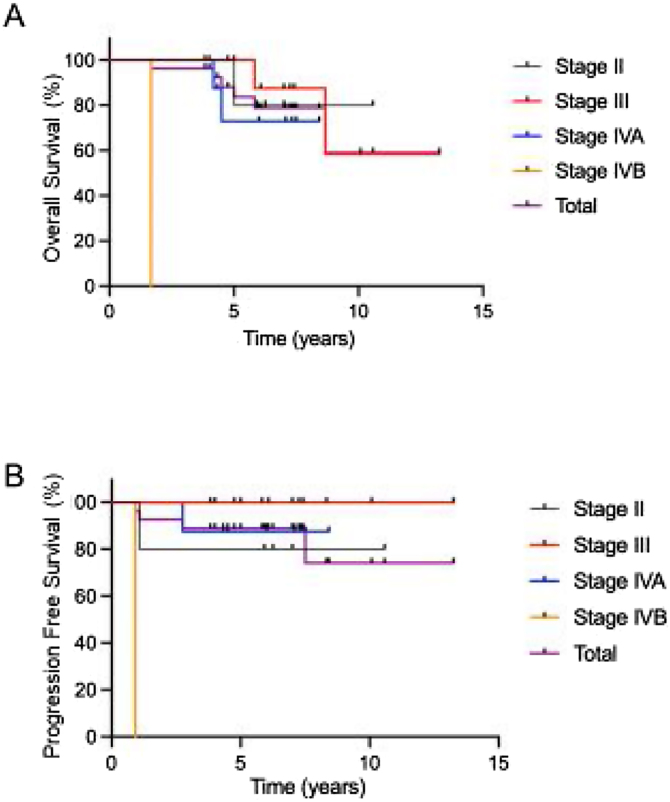
Kaplan Meier plot of overall survival and progression-free survival for patients in each stage. The 5-year OS (a) and PFS (b) of nasopharyngeal patients treated with ACRT are shown. The 5-year OS and PFS rates were 83.7% and 88.9%, respectively.

A summary of the clinical factors affecting survival rates is presented in Table 4. Only clinical stage IV was associated with a poor 5-year OS, and the 5-year PFS rate was comparable in all groups. The OS and PFS times in each stage treated with ACRT are shown in Fig. 3. The 5-year OS and PFS rates were 83.7% and 88.9%, respectively.

**Table 4:** Results of the univariate analysis of prognostic factor on 5-year OS and PFS.

Factor		Nº of patients	5-year OS	p-value	5-year PFS	p-value
Total		27	83.7		88.9	
Age	<60	15	85.7	0.698	86.7	0.720
	≥60	12	81.5		91.7	
Gender	Male	21	83.7	0.953	90.5	0.674
	Female	6	83.3		83.3	
Histology (WHO classification)	Non-Keratinizing	23	85.6	0.514	91.3	0.384
	Keratinizing	4	75		75	
T Classification	1 or 2	12	85.7	0.811	85.7	0.759
	3 or 4	15	83.5		90	
N Classification	0 or 1	14	75.5	0.347	85.7	0.623
	2 or 3	13	92.3		92.3	
Stage	II or III	18	93.3	0.047	94.4	0.199
	IV	9	64.8		77.8	
EBER	Positive	18	86.9	0.462	88.2	0.999
	Ne ative	7	75		87.5

## Discussion

Nasopharyngeal carcinoma is a chemotherapy- and radiotherapy-sensitive cancer.^9^ Increasing evidence indicates that platinum-based CCRT is the mainstay of treatment, but the optimal CCRT regimen remains unclear.^10^, ^11^, ^12^ Moreover, compliance with platinum-based CCRT is generally low. In detail, You et al. reported that 212 of 689 patients (30.8%) in the CCRT group completed three cycles of 100 mg/m^2^ cisplatin every 3 weeks with IMRT, and that 3-year OS was 92.9%.^13^ Li et al. showed that 134 of 239 (56.1%) patients completed three cycles of 100 mg/m^2^ cisplatin every 3-weeks with IMRT (median dose: 70 Gy), and that 5-year OS was 77.7%.^6^

Fuwa et al. first reported the benefits of ACRT in 2007. According to their report, the 5-year OS and PFS rates were 83% and 75%, respectively.^7^ These were comparable to the 5-year OS and PFS rates of 83.7% and 88.9%, respectively, found in our study. These rates are not inferior to those of CCRT and induction chemotherapy with CCRT.^4^, ^10^, ^12^, ^14^, ^15^, ^16^ Furthermore, one of the advantages of ACRT is its high treatment compliance.

To the best of our knowledge, five previous studies regarding ACRT for NPC have been published.^7^, ^15^, ^17^, ^18^, ^19^ There were 233 NPC patients treated by ACRT in those studies, and the median 5-year OS and PFS rates and the completion rate were 83% and 70%, and 82%, respectively. These studies basically followed the original method reported by Fuwa et al.^7^ Conversely, in the study regarding CCRT for 2,384 NPC patients,^1^ which was a meta-analysis comprising eight Randomized Controlled Trials (RCTs), showing that in terms of chemotherapy regimens, five trials used a weekly low cisplatin dose (30–40 mg/m^2^, up to 8 weeks) and three trials used a 3-weekly high cisplatin dose (80–100 mg/m^2^, three cycles during irradiation period). Regarding radiotherapy, two trials used IMRT, and the other six trials used non-IMRT techniques such as two- or three-dimensional radiotherapy. In the eight RCTs, the median 5-year OS and PFS rates and the completion rate were 74% and 59%, and 61%, respectively.

In the present study, 59%, 70%, and 11% of patients experienced grade 3 or 4 leukopenia, grade 3 or 4 mucositis, or grade 3 or 4 thrombocytopenia, respectively, but the treatment completion rate was high regardless of these adverse effects. This suggests that most adverse events were manageable, allowing patients to continue the ACRT regimen.

A disadvantage of ACRT for NPC patients is its long treatment period. It was previously reported that long duration of treatment was correlated with poor OS in head and neck squamous cell carcinoma.^20^, ^21^ In addition, both prolongation of the radiotherapy and radiation treatment breaks were correlated with loss of local control in head and neck cancer^22^, ^23^; however, patients may have enough time to overcome adverse effects in that period, as our research shows that patients can achieve not only good 5-year OS and PFS rates but also a good completion rate.

Regarding prognostic factors, only clinical stage IV disease was identified as a significant cause of poor 5-year OS in patients treated with ACRT. Although Keratinizing Squamous Cell Carcinoma (KSCC) has been reported to be correlated with poor prognosis in patients with NPC,^24^ no significant differences were found between KSCC and Non-Keratinizing Carcinoma (NKC) in our study; this was possibly due to the small number of patients enrolled in our study.

Plasma EBV-DNA is a potential biomarker for NPC progression.^25^ EBV-DNA measurement might be useful for detecting not only NPC recurrence but also early asymptomatic NPC.^26^ EBV-DNA viral load decreased to undetectable levels in all cases in this study.

A limitation of this study was the small sample size due to the rarity of NPC patients in Japan, unlike in East and Southeast China, which have a higher incidence of NPC. Further studies are warranted to confirm the clinical significance of ACRT for the treatment of NPC.

## Conclusion

This study demonstrated that ACRT for the treatment of NPC was well tolerated with acceptable efficacy. This treatment could achieve not only a high treatment completion rate, but also good 5-year OS and PFS rates.

## Ethical consideration

All procedures performed in this studies involving human participants follows the 1964 Helsinki declaration and its later amendments or comparable ethical standards. We obtained written informed consent from all individual participants included in the study.

## Funding

The authors have no funding for this study.

## Disclosure statement

All authors certify that we accept the double-blind policy of Clinical Otolaryngology.

## Conflicts of interest

The authors declare no conflicts of interest.
