# Supplementary material for: Treatment outcomes of alternating chemoradiotherapy for nasopharyngeal carcinoma: a single-center safety and efficacy study
Source: Braz J Otorhinolaryngol. 2022 Dec 28;89(3):440–6. doi: 10.1016/j.bjorl.2022.12.004 (PMC10164767; doi:10.1016/j.bjorl.2022.12.004)
Supplement: Supplementary file 1 [file mmc1.docx]

BJORL-D-22-00284_Supplementary Material

**Supplemental Table 1** Clinical characteristics of the patients (n = 10).

| **Characteristics** | | **Nº of patients** |
| --- | --- | --- |
| Age, year | Range | 42‒88 |
|  | Median | 70 |
|  | Mean | 68.5 |
| Stage | I | 1 |
|  | II |  |
|  | III | 5 |
|  | IVA | 2 |
|  | IVB | 2 |
| Sex | Male | 9 |
|  | Female | 1 |
| Histology | Non-keratinizing | 9 |
|  | Keratinizing | 1 |
| EBER ISH | Positive | 6 |
|  | Negative | 1 |
|  | NP | 3 |

NP, Not Performed.
